# Hepatorenal Syndrome: Outcome of Response to Therapy and Predictors of Survival

**DOI:** 10.1155/2015/457613

**Published:** 2015-04-23

**Authors:** Jan Heidemann, Christoph Bartels, Christoph Berssenbrügge, Hartmut Schmidt, Tobias Meister

**Affiliations:** ^1^Department of Gastroenterology, Klinikum Bielefeld, 33604 Bielefeld, Germany; ^2^Department of Medicine B, University of Münster, 48149 Münster, Germany; ^3^Department of Transplantation Medicine, University of Münster, 48149 Münster, Germany; ^4^Department of Medicine II, HELIOS Albert-Schweitzer Hospital, Academic Teaching Hospital of Georg-August-University of Göttingen, 37154 Northeim, Germany

## Abstract

*Aim*. Treatment of hepatorenal syndrome (HRS) in patients with liver cirrhosis is still challenging and characterized by a very high mortality. This study aimed to delineate treatment patterns and clinical outcomes of patients with HRS intravenously treated with terlipressin. *Methods*. In this retrospective single-center cohort study, 119 patients (median [IQR]; 56.50 [50.75–63.00] years of age) with HRS were included. All patients were treated with terlipressin and human albumin intravenously. Those with response to treatment (*n* = 65) were compared to the patient cohort without improvement (*n* = 54). Patient characteristics and clinical parameters (Child stage, ascites, hepatic encephalopathy, HRS type I/II, and initial MELD score) were retrieved. Univariate analysis of factors influencing the success of terlipressin therapy and Cox regression analysis of factors influencing survival was carried out. *Results*. One-month survival was significantly longer in the group of responders (*p* = 0.048). Cox regression analysis identified age [Hazard ratio, 95% confidence interval (CI); 1.05, 1.01–1.09, resp.], alcohol abuse [HR 3.05, 95% CI 1.11–8.38], duration of treatment [HR 0.92, 95% CI 0.88–0.96], and MELD score [HR 1.08, 95% CI 1.02–1.14] to be independent predictors of survival. *Conclusions*. Survival of HRS patients after treatment depends on age, etiology of liver disease, and the duration of treatment.

## 1. Introduction

Hepatorenal syndrome (HRS) is defined as a potentially reversible kidney failure in patients with liver cirrhosis, acute liver failure, or alcoholic hepatitis [1, 2]. Due to its very high short-term mortality [3], HRS is a life-threatening condition that has to be diagnosed and treated rapidly in order to improve the patient's clinical outcome. The pathogenesis of HRS comprises portal hypertension with impaired kidney perfusion by vasoconstrictor endogenous mediators (including vasopressin, noradrenalin, and renin/angiotensin), leading to oliguria, very low renal sodium excretion (<10 mmol/L), and water retention [4]. Further diagnostic criteria include but are not restricted to creatinine serum concentrations rising above 1.5 mg/dL and clinical exclusion of other causes of acute kidney failure, structural kidney disease, shock, dehydration, and nephrotoxic medication [4, 5]. Systemic infections are potentially predisposing causes in some patients with HRS. About 40% of patients with liver cirrhosis, ascites, and normal retention parameters will develop HRS within five years [6].

Type I HRS is a subtype with poor prognosis [7] which develops rapidly, showing doubling of the serum creatinine concentration within two weeks, whereas type II HRS patients have slower rising renal retention parameters and a propensity to develop ascites. Type I HRS patients have a mortality of 50% two weeks after diagnosis, approaching up to 100% within months [8]. The median survival of untreated type I HRS was calculated to be approximately 11 days, with a survival probability of 25% after 30 days [4]. Patients with type II HRS show a lower mortality rate with the median survival being approximately six months.

A number of interventional studies indicate that a combined intravenous therapy with vasopressor drugs such as terlipressin and human albumin improves kidney function and enhances survival in type I HRS patients [9–12]. Pretransplantation therapy with albumin and terlipressin was shown to improve the postoperative course in patients undergoing liver transplantation [13].

Combined terlipressin/albumin treatment has emerged as standard medical treatment for patients with HRS type I in the last years. Guidelines released by the German Society of Gastroenterology (Deutsche Gesellschaft für Verdauungs- und Stoffwechselerkrankungen (DGVS)) in 2011 recommend combined terlipressin/albumin i.v. treatment in patients with HRS type I [14] based on data showing improved short-time survival in these patients. In type II HRS patients, however, efficacy of combined terlipressin/albumin treatment has not been finally determined.

In this retrospective, single-center analysis of a tertiary care center (University Hospital of Münster, Departments of Gastroenterology and Transplantation Medicine), we aimed to delineate treatment patterns, doses, and clinical outcomes of patients with HRS intravenously treated with the vasopressin analogue terlipressin. Furthermore, we conducted regression analysis in order to identify predictors of survival in patients with HRS.

## 2. Materials and Methods

The study was conducted as a retrospective, single-center analysis using the International Classification of Diseases (ICD) endorsed by the World Health Organization (WHO). From January 2005 to March 2014, inpatients at the Departments of Gastroenterology and Transplantation Medicine, University of Münster, were coded as hepatorenal syndrome (ICD code K76.7). A total of 119 complete files of patients treated from HRS were retrieved and were appropriate for retrospective analysis. HRS patients with response to treatment were compared to the patient cohort without improvement. Baseline characteristics (e.g., age, gender, and underlying disease) were retrieved. The present study has been performed in accordance with the ethical standards laid down in the 2000 Declaration of Helsinki.

### 2.1. Inclusion and Exclusion Criteria and Definition of Hepatorenal Syndrome

For this study, complete patients files coded for ICD code K76.7 (hepatorenal syndrome) were screened for inclusion in this study. Reasons for exclusion of analysis were incomplete patient files, patients not having received terlipressin treatment, multiple files per patient, or miscoding/misclassification with regard to hepatorenal syndrome as classified by the criteria established by Salerno et al. in 2007 [5].

Retrospectively, patient files were assessed for the presence of hepatorenal syndrome types I or II using the modified Salerno criteria; all patients had to present with serum creatinine of >1.5 mg/dL, and patients with other reasons for acute renal dysfunction (e.g., SIRS or sepsis) were excluded.

### 2.2. Definition of Response to HRS Therapy

The treatment response was defined as any serum creatinine of 1.5 mg/dL or below after therapy with terlipressin was commenced. In all patients, the retention parameters serum creatinine and urea were measured the first day after treatment initiation. Both retention parameters were then monitored every 48–72 hrs.

### 2.3. Statistical Analysis

Descriptive analysis was used to document the demographic and clinical data of the patients. Data were analyzed using SPSS 17.0 (Chicago, IL, USA). Results are expressed as means ± standard deviation or medians [interquartile range]. Comparisons between groups were performed by using the Mann-Whitney* U*-test or two-sided *χ* ^2^ test being appropriate for the detection of statistical significance. Univariate analysis for identifying possible predictors of response to HRS therapy was performed. A *p* value < 0.05 was considered statistically significant. Variables with a significant association in the univariate analysis were analyzed with multivariate binary logistic regression for identifying independent factors. The Kaplan-Meier estimator method was applied to calculate median survival, and the log-rank test was used for assessment of statistical significance. Multivariate analysis of factors influencing one-month survival was carried out using the Cox regression model.

## 3. Results

Within the study period of nine years, 65 HRS patients (55%) with response to terlipressin therapy were compared to a cohort of 54 patients (45%) without adequate response. The median age and gender distribution were similar in both groups. No statistically significant difference could be observed in terms of the underlying disease. When considering the distribution of the Child stage, significantly more patients with Child A cirrhosis can be found in the group of responders (*p* = 0.042), while in the cohort of nonresponders to terlipressin, significantly more patients showing Child B cirrhosis are observed (*p* = 0.007). A detailed description of the baseline characteristics is presented in Table 1.

In univariate analysis, only initial serum protein was statistically different between the two groups (responders: 5.84 ± 1.27 g/L versus nonresponders: 5.30 ± 1.47 g/L; *p* = 0.04) (Table 2).

**Figure 1:**
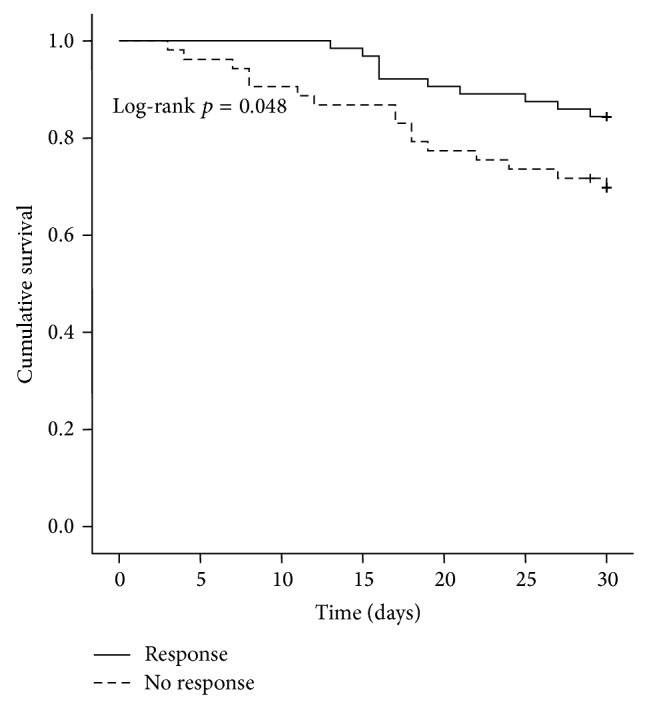
Kaplan-Meier survival analysis of the patient cohort: mean short-term survival was significantly longer in the responder group compared with nonresponders to terlipressin therapy: 28.4 days [95% CI 27.3–29.4] versus 25.6 days [95% CI 23.3–27.8] (responder versus nonresponder group, log-rank test, and *p* = 0.048).

**Figure 2:**
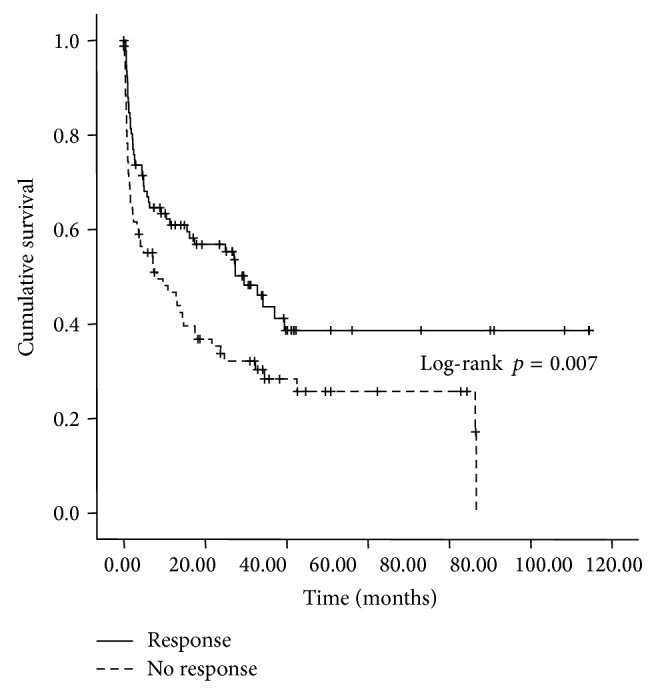
Kaplan-Meier survival analysis of the patient cohort: median overall survival was significantly longer in the responder group compared with nonresponders to terlipressin therapy: 29 months [95% CI 20.7–38.4] versus 8 months [95% CI 0.0–16.3] (treatment responders versus nonresponder group, log-rank test, *p* = 0.007).

Kaplan-Meier calculation indicates that response to terlipressin therapy in HRS patients is a significant predictor of survival. The mean short-time survival (30 days) for the responder group is significantly longer compared to the group of nonresponders (28.4 days [95% CI 27.3–29.4] versus 25.6 days [95% CI 23.3–27.8], *p* = 0.048, Figure 1). The median overall survival of responding patients was 29 [95% CI 20.69–38.44] months compared to 8 months [95% CI 0.0–16.33] for the nonresponding group (*p* = 0.007, Figure 2).

**Figure 3:**
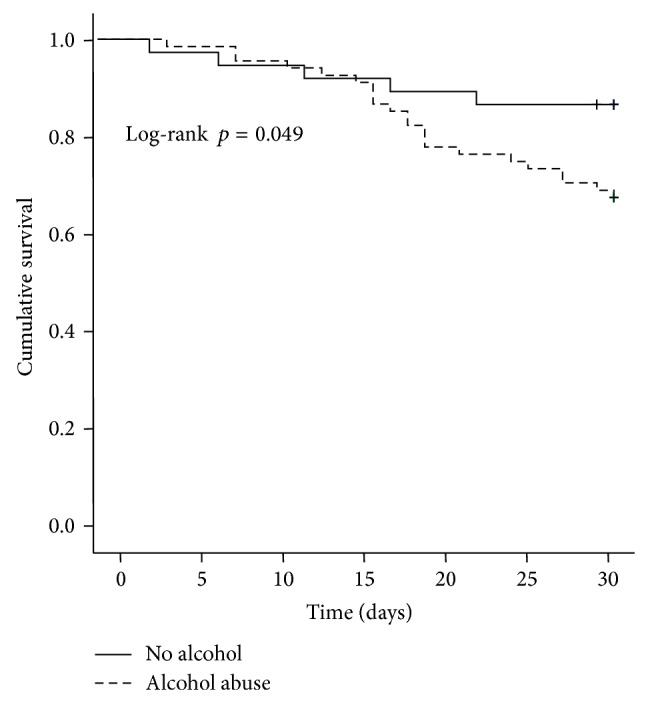
Kaplan-Meier survival analysis of the patient cohort: mean short-term survival was significantly longer in the group without alcohol abuse compared with abusers: 27.9 days [95% CI 26.1–29.8] versus 26.7 days [95% CI 25.2–28.1] (alcohol abuse versus nonalcohol abuse, log-rank test, *p* = 0.049).

**Figure 4:**
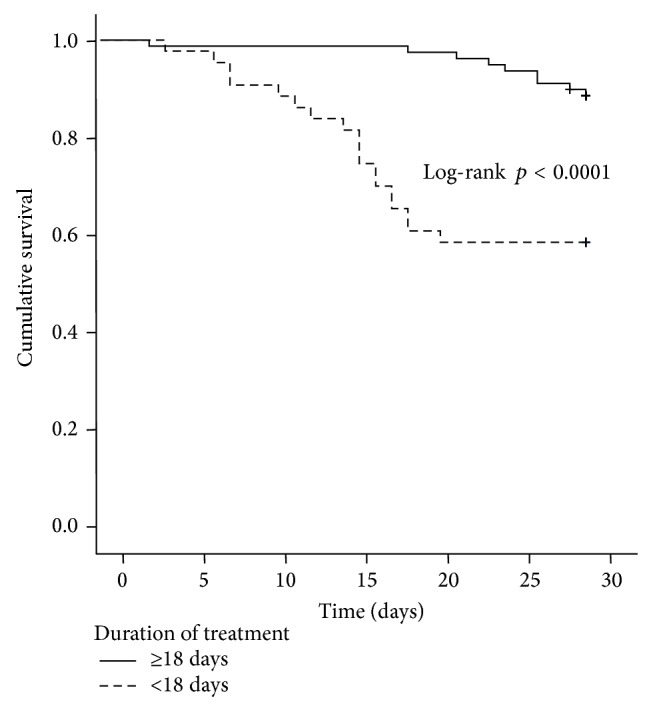
Kaplan-Meier survival analysis of the patient cohort: mean short-term survival was significantly longer in group with treatment duration >18 days compared with those less than 18 days treated: 29.2 days [95% CI 28.4–30.0] versus 23.4 days [95% CI 20.9–25.9] (treatment days ≥ 18 versus treatment days < 18 days, log-rank test, *p* < 0.0001).

**Figure 5:**
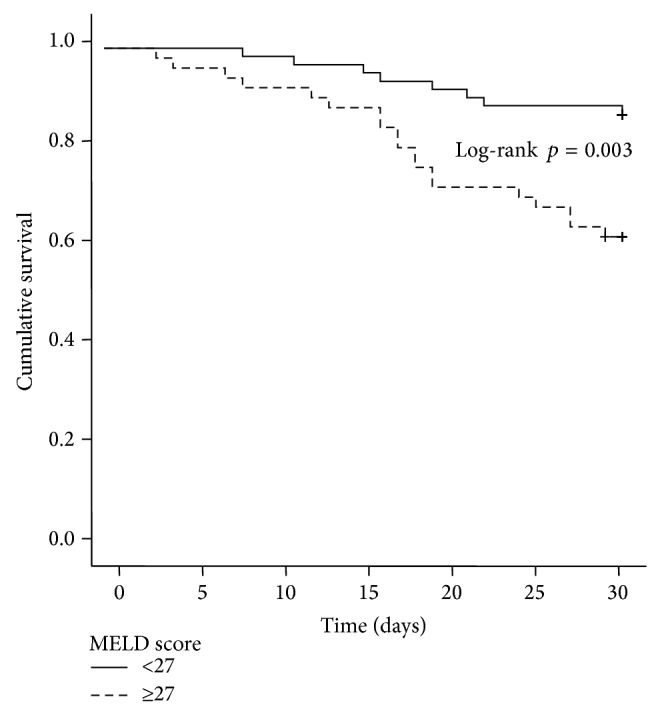
Kaplan-Meier survival analysis of the patient cohort: mean short-term survival was significantly longer in patients with MELD score less than 27 compared with those having a MELD score >27: 28.5 days [95% CI 27.3–29.7] versus 25.4 days [95% CI 23.4–27.5] (MELD score < 27 versus MELD score ≥ 27, log-rank test, *p* = 0.003).

Patients with alcohol abuse had a significant lower short-time survival compared to those without alcohol problems (mean survival, 26.7 days [95% CI 25.2–28.1] versus 27.9 days [95% CI 26.1–29.8], *p* = 0.049, Figure 3). Short-term survival was significantly prolonged in patients more than 18 days of hospital treatment (duration ≥18 days: 29.2 days [95% CI 28.4–30.0] versus duration <18 days 23.4 days [95% CI 20.9–25.9], *p* < 0.0001, Figure 4) and in patients with an initial MELD score less than 27 (28.5 days [95% CI 27.3–29.7] versus 25.4 days [95% CI 23.4–27.5], *p* = 0.003, Figure 5).

In the Cox proportional hazard model, only age (HR 1.05 [95% CI 1.005–1.093]), alcohol abuse (HR 3.05, [95% CI 1.111–8.384]), duration of treatment (HR 0.92 [95% CI 0.875–0.964]) and MELD score (HR 1.08 [95% CI 1.019–1.141]) proved to be independent prognostic survival factors (Table 3).

## 4. Discussion

Cirrhotic patients with portal hypertension are at high risk to develop a multitude of renal dysfunction patterns, including paracentesis-induced circulatory dysfunction (PICD) and fully established hepatorenal syndrome (HRS). Leithead et al. have extensively reviewed the recent progress in the pathophysiology and treatment of portal hypertension-related renal dysfunction, which occurs as a multifactorial pathophysiological sequence on the background of profound circulatory and neurohumoral alterations in cirrhotic patients [15]. Both PICD and HRS are associated with increased morbidity and mortality in cirrhotic patients [6]. Acute renal dysfunction in cirrhotic patients is often observed to occur as a consequence of systemic inflammatory responses such as infection or sepsis. Up to 40% cirrhotic patients show circulating bacterial DNA [16] and elevated levels of lipopolysaccharide binding protein [17] as markers of clinically inapparent bacterial translocation from the intestine, which is thought to render patients more susceptible to renal failure.

Therefore, the prevention of any renal further impairment in cirrhotic patients with portal hypertension-related renal dysfunction is of utmost importance for prognosis and survival. These include avoidance of nephrotoxic drugs (e.g., NSAID and ACE-Inhibitors), prevention and treatment of infection (including spontaneous bacterial peritonitis), prevention and treatment of gastrointestinal bleeding, avoidance of large volume paracentesis without albumin replenishment [18, 19], and management of sodium and water retention [19].

Therapeutic agents tested for the prevention of hepatorenal syndrome include vasoactive compounds, human albumin infusion, antibacterial substances such as rifaximin [20] or norfloxacin [21, 22], and specific enteral nutrition containing the antioxidative glutathione precursor N-acetylcysteine [23], among other approaches.

Despite a small randomized trial which indicates that prophylaxis with oral pentoxifylline, an oral phosphodiesterase inhibitor, was able to prevent HRS in some patients with alcoholic steatohepatitis [24], this substance did not improve survival in patients with advanced liver cirrhosis [25]. Treatment of cirrhotic and ascitic patients with the directly vasoconstrictive drug midodrine was clearly inferior to human albumin infusion treatment in the prevention of paracentesis-induced circulatory dysfunction and hepatorenal syndrome [26].

Based on recommendations endorsed by the leading societies of gastroenterology and hepatology [27], combined terlipressin/albumin treatment has been widely accepted as standard medical treatment for patients with acute HRS type I shortly after emergency admission. A very recent study indicates that combined albumin/terlipressin treatment appears to be safe and effective in patients presenting with acute hepatorenal syndrome associated with sepsis, further supporting early administration of this treatment [28]. Of note, terlipressin is not available in the USA and Canada up to now [29].

The criteria of when to initiate terlipressin therapy and how to judge sufficient treatment response are poorly defined. In many cirrhotic patients presenting with acute kidney failure, preclinical creatinine levels are rarely available, which leads to speculation about how quickly deterioration of renal function occurred. Furthermore, it has been criticized that the diagnosis of HRS is based on a rigid cutoff value of serum creatinine (1.5 mg/dL), because creatinine synthesis in patients is known to vary widely with regard to cachexia/muscle mass, ethnicity, gender, and age. Furthermore, it was shown that, in cirrhotic patients, serum creatinine levels are falsely low due to reduced creatinine production in liver and wasting muscles, as well as increased renal tubular secretion despite the fact that the actual glomerular filtration rate is low [30]. Moreover, the criteria defining an adequate response to treatment with terlipressin are still a matter of debate among researchers and clinicians. With regard to treatment response, our patients were judged by a sheer drop in serum creatinine levels to or below 1.5 mg/dL, whereas other groups were advocated to measure recovering daily rates of diuresis or rising renal sodium excretion [3, 31]. However, in our retrospective analysis of patient files, renal sodium excretion rates and daily rates of diuresis were only rarely recorded systematically. This may be caused by a high proportion of alcohol-associated disease and therefore possibly lower patient compliance which adequately mirrors clinical reality in the absence of prospective data.

Overall treatment response in our patient series, as judged by dropping creatinine values to or below 1.5 mg/dL was approximately 55% overall, which is in well accordance with previous studies which have indicated terlipressin treatment response in 40–60% of HRS patients [32]. Interestingly, and in contrast to previous observations, we were unable to identify more HRS type I patients than type II patients in the group of responders. The significance of this finding is unclear till now, and it can be speculated that the differentiation between HRS types I and II is erroneous according to the patient files retrospectively analyzed, with possibly underpowered study design. In a very recent retrospective study on patients with hepatorenal syndrome, the one-month mortality was not statistically different between HRS type I and II patients [33].

While short-term survival in acute HRS (i.e., type I) patients widely depends on acute clinical measures such as terlipressin/albumin treatment, calculated hydration, and differential diuretic therapy, long-term survival in patients with HRS is depending on the restitution of liver function. Therefore, liver transplantation is considered to be the first line treatment for both types of HRS [34]. The five-year survival rate after liver transplantation for HRS was reported to be 60%, with a postoperative course known to be more complicated as compared to patients with normal kidney parameters [35]. Transjugular portosystemic stenting (TIPS) will improve renal function and normalize systemic endogenous vasopressor levels in some patients [36], but many patients with HRS type I are not qualified for this procedure due to hepatic encephalopathy or impaired liver function [37]. A large meta-analysis comparing 305 HRS patients treated by TIPS procedure versus paracentesis alone has shown superior survival figures in patients receiving TIPS treatment as compared to large volume paracentesis [5]. Both TIPS and hemodialysis are considered bridging strategies for some patients until liver transplantation can be achieved. Studies exploring the efficacy of liver support dialysis including the molecular absorbent recirculating system (MARS) in type HRS I patients with cirrhosis and ascites have suggested beneficial effects regarding improvement of renal function and systemic hemodynamic parameters; however, current data reveal tendencies but with no significance [38, 39].

In our patient series, short-term survival (30 days) was significantly higher in terlipressin responders versus nonresponders (28.4 versus 25.6 days, *p* = 0.048). Therefore, it appears that acute response to terlipressin treatment is a valid predictor of higher short-term survival in HRS patients treated with terlipressin, irrespective of which HRS type was predominant (Figure 1). Using Kaplan-Meier estimation of overall survival, responders had a median survival of 29 months, whereas nonresponders had a considerably less favorable prognosis (8 months, *p* = 0.007) (Figure 2).

Interestingly, patients with Child stage B cirrhosis had a lower likelihood to respond to therapy, leading to the assumption that other renoparenchymal disorders not responsive to terlipressin treatment (e.g., diabetic nephropathy) might have mimicked hepatorenal syndrome in some patients with concomitant liver cirrhosis (Table 1). This phenomenon may be explained by the current understanding that the more “renal hits” in advanced liver disease occur, the less functional renal capacity will remain. This is represented by the term hepatorenal disease, which over time results in decreasing functional renal reserve capacity due to increasing irreversible renal damage.

Finally, Cox regression analysis identified the factors age, alcohol abuse, shorter duration of therapy, and MELD score as independent variables worsening the probability of survival in patients with HRS (Table 3). As reflected by our patient cohort, older age in general is associated with lower rates of recovery after organ failure [40, 41]. Patients with a history of alcohol abuse or ongoing alcohol addiction also have a more than three times higher probability to die within 30 days, as compared to the overall study population (HR 3.05, 95% CI 1.111–8.384, *p* = 0.031, Table 3). Patients with ongoing alcoholism are prone to malnutrition, micronutrient deficiency [42], concomitant nicotine and medications abuse [43], and low treatment compliance, all of which may have influenced the very drastic survival disadvantage observed in our patient cohort (Table 3). Longer duration of terlipressin treatment, however, was identified as an independent predictor of better one-month survival (HR 0.92, 95% CI 0.875–0.964, *p* = 0.001, Table 3), and early discontinuation of the drug occurred in 8 out of 119 patients (three or less days of terlipressin treatment: 6.1%). The optimal cutoff value for treatment duration as retrieved by ROC analysis was more than 18 days in our patient cohort with significantly longer survival compared to those who were treated for less than 18 days (29.2 days [95% CI 28.4–30.0] versus 23.4 days [95% CI 20.9–25.9], log-rank test, *p* < 0.0001, Figure 4).

Finally, corroborated by the results from earlier studies, a higher MELD score was an independent predictor of lower one-month survival (HR 1.08, 95% CI 1.019–1.141, *P* = 0.009, Table 3), albeit this signal was not as robust as one could have expected from initial studies on MELD scores and associated survival [44, 45].

## 5. Conclusions

In our patient analysis, many patients with hepatorenal syndrome were treated very early with combined albumin and terlipressin. Treatment response was approximately 55%, as expected from older studies. Our data suggest that older age, higher Child stage, alcohol abuse, and higher initial MELD score are clinical parameters associated with less favorable patient outcomes, whereas the differentiation between HRS types I and II did not influence treatment response rates in our patient series. Moreover, patients showing no sufficient treatment response show significantly higher one-month mortality than patients with terlipressin response. Finally, patients responding to terlipressin therapy have a significantly higher estimated median survival as compared to nonresponders. Age, duration of treatment, MELD score, and alcohol abuse are independent predictors of short-term survival. Given the notion that hepatorenal syndrome is today widely regarded as a potentially preventable condition, prophylaxis of any causes that precipitate of renal dysfunction should be the focus of patient care for cirrhotic patients.

## 6. Limitations

We acknowledge that our patient cohort was rather small and possible patient selection bias from a single center and misclassification or information bias as a result of the retrospective study design might impact the veracity of the findings of our study. Nevertheless, our study adds valuable new findings and endorses established knowledge in the identification of predictors of successful response to HRS therapy and survival in cirrhotic patients for better future patient care. Clearly, further studies with prospective, multicenter design will be needed in order to more closely define the predictors of treatment response and survival in patients with HRS.

## Figures and Tables

**Table 1:** Baseline characteristics of the patient cohort.

Variable	Response	No response	*p* value
Number of patients	65 (54%)	54 (45%)	
Age, median (IQR)	56 [51–62]	59 [50.5–63]	0.325
Age range	25–78	25–74	
Gender (m/f)	44/21	37/17	0.580
Etiology, *N* ^#^			
Alcohol abuse	46	31	0.130
Hepatitis B	0	3	0.055
Hepatitis C	3	6	0.184
AIH	1	0	0.362
Hemochromatosis	3	1	0.407
PBC/PSC	2	5	0.155
Cryptogenic	11	9	0.970
Child stage, *N*			0.693
A	20	8	**0.042**
B	20	30	**0.007**
C	25	16	0.404
MELD score, median (IQR)	26 [19–32]	26 [21–33]	0.505

#: more than one etiology possible.

**Table 2:** Univariate analysis of the patient cohort.

Variable	Response	No response	*p* value
HRS type 1/2, *N*	47/18	45/9	0.165
Ascites, grade 1/2/3	9/38/18	11/25/16	0.466
Treatment, median days (IRQ)	9 [6–14]	10.5 [5–19]	0.350
HE, grade 1/2/3	46/15/3	39/14/1	0.699
Serum protein, g/L	5.84 ± 1.27	5.30 ± 1.47	**0.040**
INR	1.60 ± 0.52	1.64 ± 0.44	0.698
Serum sodium (mmol/L)	133.60 ± 6.21	133.12 ± 5.73	0.714
Serum potassium (mmol/L)	4.34 ± 0.80	4.22 ± 0.71	0.381
Serum creatinine (mg/dL)	2.79 ± 1.24	3.07 ± 1.47	0.207
Urea (mg/dL)	55.30 ± 24.68	67.47 ± 51.59	0.189
Bilirubin (mg/dL)	8.12 ± 10.23	10.59 ± 12.29	0.901
Terlipressin dose (mg)	26.43 ± 30.86	32.11 ± 31.57	0.450
Albumin dose (g)	266.26 ± 236.31	298.14 ± 252.02	0.612

HE: hepatic encephalopathy; INR: international normalized ratio.

**Table 3:** Cox regression analysis and predictors of one-month mortality.

Variable	HR	95% CI	*p* value
Age	1.05	1.005–1.093	**0.027**
alcohol abuse	3.05	1.111–8.384	**0.031**
duration of therapy	0.92	0.875–0.964	**0.001**
response to therapy	0.48	0.199–1.146	0.098
MELD score	1.08	1.019–1.141	**0.009**
